# Tageskliniken zentral und/oder disloziert. Ein Standardelement der kinder- und jugendpsychiatrischen Versorgung

**DOI:** 10.1007/s40211-022-00439-8

**Published:** 2022-11-08

**Authors:** Dina Weindl, David Koller, Martin Kostial, Karin Zajec, Judith Noske

**Affiliations:** 1 Abteilung für Kinder- und Jugendpsychiatrie und Psychotherapie, LK Baden-Mödling am Standort Hinterbrühl, Hinterbrühl, Österreich; 2 University of Vienna, Wien, Österreich; 3 Ambulanz und Tagesklinik für Kinder- und Jugendpsychiatrie, LKH Graz Süd-West am Standort Leoben, Leoben, Österreich; 4 Institut für psychosoziale Medizin, Psychotherapie und Kindheitsforschung, Karl Landsteiner Gesellschaft, St. Pölten, Österreich

**Keywords:** Behandlungsbausteine, Strukturqualitätskriterien, Raumbedarf, Transdisziplinarität, Regionalität, Tagesklinik, Kinder- und Jugendpsychiatrie, Treatment modules, Structural quality criteria, Space requirements, Transdisciplinary, Regionality, Day care clinic, Child and adolescent psychiatry

## Abstract

**Hintergrund:**

Tageskliniken als fachspezifische Versorgungseinheiten der kinder- und jugendpsychiatrischen Versorgung in Österreich stellen einen wichtigen Behandlungsbaustein für personenorientierte Behandlungsangebote dar. Neben zahlreichen Vorteilen, stellen sie jedoch auch höhere Anforderungen an die jungen Patient:innen und deren Angehörige.

**Methodik:**

In der vorliegenden Arbeit wird die in Österreich etablierte tagesklinische Versorgungsstruktur, welche im Österreichischen Strukturplan Gesundheit festgehalten ist, dargestellt. Dabei wird ein besonderes Augenmerk auf die Strukturqualitätskriterien, welche sich am Konzept der Leistungsorientierten Krankenhausfinanzierung orientieren, gelegt.

**Ergebnisse:**

Es zeigt sich eine hohe Anforderung und Notwendigkeit zur Bereitschaft zu transdisziplinären, multimodalen Behandlungskonzepten und -formen, sowie ein großer räumlicher Ressourcenbedarf. Die Etablierung von mehreren tagesklinischen Gruppen an einem Standort finden sich im Konzept der Leistungsorientierten Krankenhausfinanzierung nicht wieder und es muss zur Orientierung auf andere Quellen verwiesen werden. Ebenso verhält es sich mit dem benötigten Raumbedarf.

**Schlussfolgerung:**

Zur Sicherstellung und Evaluation der Behandlungsqualität werden auf patient:innenbezogener als auch team- beziehungsweise organisationsbezogener Ebene Empfehlungen ausgesprochen. Die Arbeitsgruppe Tagesklinik der Österreichischen Gesellschaft für Kinder- und Jugendpsychiatrie, Psychosomatik und Psychotherapie empfiehlt neben der regionalisierten und wohnortnahen Versorgung und der Integration der Tagesklinik in die Gemeinde eine Behandlungsstruktur, welche durch Transdisziplinarität sowie Partizipation geprägt ist. Die Vernetzung mit anderen Versorgungsbereichen, sowie die Nutzung sozialpsychiatrischer Netzwerke ist unerlässlich. Regionale Besonderheiten sollten Berücksichtigung finden und spezialisierte, themenspezifische tagesklinische Gruppen sollten in weiteren Planungen und Konzepten vermehrt Eingang finden.

## Abstract (en)

**Background:**

Day-care clinics as specialized care units of child and adolescent psychiatric care in Austria represent an important component for person-orientated treatment offers. In addition to numerous advantages, they also put higher demands on the young patients and their relatives.

**Methods:**

The Austrian structure for day-care settings is recorded in the Austrian structure plan for health. Therefore, particular attention is paid to structural quality criteria, which are based on the Austrian concept of performance-oriented hospital financing.

**Results:**

A high demand and need for readiness for transdisciplinary, multimodal treatment concepts and forms is discussed. It becomes ovious that there is a need for large spatial resource requirements. The establishment of several day clinic groups at one location and spatial requirements cannot be found in the concept of performance-oriented hospital financing.

**Conclusion:**

To ensure and evaluate the quality of treatment, recommendations are made on a patient-related and team- or organization-related level. The workgroup “day-care-clinic” of the Austrian Society for Child and Adolescent Psychiatry, Psychosomatics and Psychotherapy recommends a treatment structure characterized by transdisciplinarity and participation. Further, it regionalized and local access and the integration of the day clinic into the community is necessary. Networking with other care structures and using social-psychiatric networks is essential. Regional characteristics should be taken into account and specialized, topic-specific day clinic groups should be increasingly included in further planning and concepts.

## Einleitung

Tageskliniken für den Bereich der Kinder- und Jugendpsychiatrie (KJP) wurden im internationalen Rahmen als Standardelemente einer fachspezifischen Versorgung entwickelt. Tageskliniken sind ein wichtiger Baustein in der flächendeckenden kinder- und jugendpsychiatrischen Versorgung, die zwischen dem ambulanten und stationären Bereich angesiedelt sind. Sie bieten den Vorteil einer wohnortnahen Behandlung [1], die psychisch kranken Kindern und Jugendlichen ein vielseitiges therapeutisches und pädagogisches Angebot unter Beibehaltung des familiären Settings ermöglicht, und so einer nicht zu unterschätzenden Hospitalisierungsgefahr entgegenwirken [2]. Gleichzeitig stellt eine tagesklinische Behandlung für alle Beteiligten ein hohes Anforderungsniveau. Es zeigte sich in der Vergangenheit, dass vor allem tagesklinische Patient:innen tendenziell jünger und aus vollständigen Familien kamen [3, 4]. Dabei spielt die elterliche Kooperation eine erhebliche Rolle [5] und kann die tagesklinische Behandlung deutlich beeinflussen. Das tagesklinische Setting kann bei Erfüllung des hohen Anforderungsprofils einen geschützten Raum für Übung und psychische Entwicklung darstellen [6].

## Versorgungslage in Österreich

In Österreich ist derzeit das Verhältnis einer tagesklinischen zu stationären Versorgung 30 %:70 %, mit 155:365 Plätzen (Stand 2019) [7]. Diese Versorgung erfolgt in 22 Tageskliniken und 15 KJP Abteilungen österreichweit [8]. Die tagesklinische Versorgung wird im Österreichischen Strukturplan Gesundheit (ÖSG) [9] unter „Akut stationäre inklusive tagesklinische Versorgung“ abgehandelt und stellt eine wichtige Ebene der nach Art. 15aB-VG geforderten integrativen Versorgungsplanung dar [10]. Als einer der Versorgungsgrundsätze wird eine tagesklinische, vor einer vollstationären Behandlung genannt. Die konkrete Umsetzung der im ÖSG entwickelten Richtlinien erfolgt entsprechend der regionalen Strukturpläne der Bundesländer z. B. [11].

Kinder- und jugendpsychiatrische Tageskliniken werden in zwei Varianten geführt:

- intramural als Teil einer bettenführenden Abteilung
- extramural in Verbindung mit extramuralen sozialpsychiatrischen Diensten.

In beiden Fällen bedarf es einer Vorschaltambulanz, in der die Aufnahmeindikation gestellt wird und die Patient:innen für eine Aufnahme vorbereitet werden.

Der Versorgungsauftrag beinhaltet eine ambulante Fachversorgung sämtlicher kinder- und jugendpsychischer Krankheiten und Verhaltensauffälligkeiten für die Altersspanne von 0–18 Jahren, einschließlich entwicklungsbedingter psychischer Störungen. Behandlungsbausteine im Behandlungsprozess sind unter anderem Krisenintervention, Diagnostik und Therapie, Nachsorge, sowie die interdisziplinäre Zusammenarbeit mit angrenzenden Fachbereichen (Pädiatrie, Erwachsenenpsychiatrie im Rahmen der Transition), mit Einrichtungen im Sozialbereich, Kinder- und Jugendhilfe, Patiente:innenanwaltschaft und die Vernetzung mit anderen Helfer:innensystemen [9].

Im Unterschied zu einem vollstationären Setting müssen im ambulanten Setting folgende Voraussetzungen erfüllt sein:

- Mögliche akute selbst- oder fremdgefährdende Krisen dürfen den Rahmen der Tagesklinik nicht überfordern.
- Die Patient:innen müssen in der Lage sein die täglichen Wechsel der Settings zu bewältigen (häusliche Umgebung, Tagesklinik, ev. Schule).
- Bezugspersonen sind in einem hohen Ausmaß gefordert, die Behandlung aktiv zu unterstützen.
- Die Fahrt zur Tagesklinik sowie die Heimfahrt muss bewältigbar sein und sollte dabei 60 Minuten nicht überschreiten [9].

## Strukturqualitätskriterien

Die Strukturqualitätskriterien für Tageskliniken und Ambulanzen basieren in Österreich auf dem Konzept der Leistungsorientierten Krankenhausfinanzierung (LKF Modell) [12]. Wenn Aspekte, die im LKF Modell keinen Niederschlag gefunden haben (Notwendigkeit einer Gruppenteilung, Ausfallszeiten, Berücksichtigung von Leitungsaufgaben, …) zusätzlich zu gewichten sind, muss auf die in Deutschland angewandte Personalausstattung Psychiatrie und Psychosomatik Richtlinie (PPP-RL) zurückgegriffen werden [13].

Im ÖSG finden sich die Planungsrichtwerte für die tagesklinische Versorgung (Planungshorizont 2025). Derzeit ist eine ambulante KJP-Einheit mit 10 Behandlungsplätzen pro 250.000 Einwohner:innen vorgesehen. Dies ergibt sich durch die Platzmesszahl (PMZ), welche tagesklinische als auch ambulante Betreuungsplätze inkludiert und im ÖSG mit 0,04 pro 1000 Einwohner:innen festgelegt ist [8]. Die Mindestanzahl an Behandlungsplätzen einer tagesklinischen Einheit darf lt. LKF fünf Behandlungsplätze nicht unterschreiten [12].

Die Patient:innen in der tagesklinischen Behandlung haben eine tägliche Anwesenheitspflicht von mindestens sechs Stunden (tagesklinische Behandlung) bzw. von mindestens vier Stunden (tagesstrukturierende Behandlung). In begründeten Fällen kann ausnahmsweise von der täglichen Anwesenheitspflicht Abstand genommen werden Die Abrechnung erfolgt über die Berechnungsbasis ambulanter Pauschalgruppen gemäß des LKF-Modelles (Behandlungspauschale AMG 20.07 für tagesklinische Leistungen; Behandlungspauschale AMG 20.08 für tagesstrukturierte Leistungen) [12].

### Personalbedarf

Der Personalbedarf von KJP Tageskliniken wird im LKF-System seit 2018 im spitalsambulanten Bereich geführt. Die Funktionseinheit einer ambulanten tagesklinischen oder ambulanten tagesstrukturierenden Behandlung muss Teil einer bettenführenden kinder- und jugendpsychiatrischen Abteilung sein.

Die Behandlung in der KJP Versorgung verfolgt einen multimodalen, transdisziplinären Ansatz [14]. Gearbeitet wird in multiprofessionellen Teams, bestehend aus Fachärzt:innen für Kinder- und Jugendpsychiatrie, Klinischen Psycholog:innen, Psychotherapeut:innen, Sozialpädagog:innen, Physiotherapeut:innen, Ergotherapeut:innen, Logopäd:innen, diplomierten Gesundheits- und Krankenpfleger:innen (DGKP), Sozialarbeiter:innen und weiteren Therapeut:innen. Die multimodale Behandlungsplanung lt. LKF sieht unterschiedliche Leistungen aus mindestens drei Bereichen des multiprofessionellen Teams vor, wie z. B. soziales Training, Lernförderung, Werkstätte, Arbeit mit Eltern und Bezugspersonen, Ergotherapie, Logopädie, Einzelpsychotherapie, Gruppentherapie, etc.

Da die fallbezogene Arbeit in der Tagesklinik zu einem Großteil auch von klinischen Psycholog:innen geführt werden kann, können Ärzt:innenstunden auch mit diesen besetzt werden (Tab. 1). Tageskliniken, die nicht im räumlichen Verbund mit stationären Einrichtungen stehen, stehen vor der zusätzlichen Herausforderung die permanente ärztliche Präsenz (während der Öffnungszeiten) aus dem tagesklinischen Personalkontingent abdecken zu müssen.

**Table 1:** 

	Fachärzt:innen bzw. in Ausbildung, Psycholog:innen	DGKP, Sozialpädagog: innen	Multi-professionelles Team
*Qualitativ*	Mind. 1,5 ärztl. Pers./1,0 Psy	Mind. 1,5 Pflege	Mind. 3 Qualifikationen aus den Gruppen
*Quantitativ (VZÄ je Patient:in)*	0,4	0,3	0,17
*Quantitativ (Patientin je VZÄ)*	2,5	3,3	5,89

Bei der Etablierung von mehreren tagesklinischen Gruppen, kann in Österreich auf kein bestehendes Leistungssystem zurückgegriffen werden. Für eine Gruppenaufteilung (Altersspezifität und gruppendynamische Überlegungen in Bezug auf Gruppengröße) werden keine Berechnungsgrundlagen angeführt. In Deutschland sah die am 01.01.2020 außer Kraft getretene Psychiatrie Personalverordnung (Psych-PV) in der Kategorie KJ7 (tagesklinische Behandlung KJPP) zusätzlich zu den 261 Minuten pro Patient pro Woche für Pflege und Erziehung zuzüglich einen Basiswert von 5000 Minuten pro Gruppe vor. Außerdem wurde die durchschnittliche Ausfallszeit pro Mitarbeiter mit 20 % veranschlagt und Leitungsfunktionen zusätzlich berücksichtigt. Ebenso konnte mit der Psych-PV auf den vermehrten Betreuungsaufwand bei Notwendigkeit der Gruppenteilung eingegangen werden (Basiswert Pflege und Erziehung pro Gruppe) [15].

### Raumbedarf

Kinder und Jugendliche verbringen in den Tageskliniken Zeit, die sie sonst in Kindergärten, Schulen, Ausbildungsorten, aber auch in ihrer Freizeit verbringen würden. Durch diese unterschiedlichen Anforderungen wird man mit einer (innen)architektonischen Herausforderung konfrontiert. Einerseits sollte den institutionellen Anforderungen wie Hygiene, Brandschutz und Sicherheit, und andererseits der funktionellen Ausstattung der Tagesstruktur (Schule, Arbeit und Freizeit), sowie den kinder- und jugendpsychiatrischen und therapeutischen Bedürfnissen entsprochen werden. Dabei bedarf es einer altersadäquaten Gestaltung der Umgebung mit entwicklungsfördernden architektonischen Elementen von Individualräumen und Gemeinschaftsbereichen, sowie einer Funktionalität der Gestaltungselemente für den tagesklinischen Alltag. Eine positive, vertrauensfördernde, den Genesungsprozess unterstützende Atmosphäre sollte dabei im Fokus stehen [16].

In ihrem Review zeigen Fricke et al. [17], dass zur Förderung von therapeutischen Prozessen bei Kindern und Jugendlichen unter anderem architektonische Variablen wie Tageslicht, bewegungsfördernde, orientierende Raumstrukturen, das Vermitteln des Gefühl des Willkommen-Seins, Außenbereiche und „Frei“räume benötigt. So konnten Wöckel et al. [18] zeigen, dass nach einer innenarchitektonischen Umgestaltung der KJP eine Verkürzung der Verweildauer der unfreiwillig untergebrachten Patient:innen erfolgte.

Weiters ist der enorme Raumbedarf pro behandelnder Patient:in zu beachten, der sich durch unterschiedliche Anforderungen wie architektonische Elemente, therapeutische-, funktionale- und institutionelle Anforderungen zusammensetzt. Die BAG leitender Mitarbeiter:innen des Pflege und Erziehungsdienstes KJP empfiehlt für ein tagesklinisches Setting (inkl. Personal‑, Therapie‑, Aufenthalts- und Ruheräume, sowie Sanitärräume) bei einer Patient:innenanzahl von max. 12 Personen ein Mindestnutzfläche von 55 m^2^ pro Patient:in im Innenbereich und 40 m^2^ im Außenbereich [19]. IM ÖSG finden sich lediglich wenige Hinweise bezüglich des notwendigen Raumbedarfs. Patient:innenzimmer sollen alterstufengerecht und mit geringem Gefährdungspotential ausgestattet sein und die Therapieräume für unterschiedliche Berufsgruppen multifunktional nutzbar und wenn möglich mit audiovisuellem Technikangebot ausgestattet sein [9].

### Prozessmanagement und Evaluation

Zur Sicherstellung der Behandlungsqualität empfiehlt die Arbeitsgruppe Tagesklinik der Österreichischen Gesellschaft für Kinder- und Jugendpsychiatrie, Pschosomatik und Psychotherapie (ÖGKJP) verschiedene Maßnahmen auf Patient:innen-, als auch auf Organisationsebene.

Patient:innenbezogen:

- Erstellen eines tagesklinischen Behandlungsplanes
- Durchführen einer leitlinienorientierten Diagnostik und Therapie sowie pädagogischer Angebote
- Moderieren von Übergängen zwischen den unterschiedlichen Versorgungsbereichen (ambulant, teilstationär, vollstationär)
- Regelmäßige patientenbezogene Besprechungen im multiprofessionellen Team mit Evaluation des Behandlungsprozesses
- Fallsupervision
- Arbeit mit dem sozialen System
- Vernetzung mit anderen Helfer:innensystemen

Team- und Organisationsbezogen:

- Regelmäßige Organisationsbesprechungen
- Regelmäßige Teamsupervisionen
- Teamklausurtage zur Überprüfung, Adaptation und möglichen Erweiterung der Behandlungskonzepte
- Funktionsbeschreibungen für die einzelnen Berufsgruppen unter Berücksichtigung von Interdisziplinarität

## Umsetzungsempfehlungen und Zukunftsideen

In der Umsetzung der Errichtung von Tageskliniken sollten hinsichtlich der Standortfrage die Versorgungsgrundsätze des ÖSG Berücksichtigung finden. Hier ist insbesondere die regionalisierte und wohnortnahe Versorgung und die Integration in die Gemeinde hervorzuheben [9].

Neben den oben ausgeführten Rahmenbedingungen innerhalb der tagesklinischen Einrichtung, deren konkrete Ausgestaltung insgesamt die Grundsätze von Transdisziplinarität sowie Partizipation widerspiegeln sollen, empfiehlt die ÖGKJP ein zentrales Augenmerk auf die Vernetzung mit anderen Versorgungsbereichen, sowie auf die Nutzung oder gegebenfalls Bildung regionaler sozialpsychiatrischer Netzwerke, unter Einbeziehung aller wesentlichen Anbieterstrukturen (inkl. Sozial- und Behindertenbereich, Kinder- und Jugendhilfe, Schulsystem, Arbeitsintegration, extramurale Anbieter, Beratungsstellen, Jugendzentren, Freizeiteinrichtungen, …) zu legen.

Die Tagesklinik sollte an allen fünf Werktagen der Woche in entsprechendem Ausmaß geöffnet haben, um innerhalb des Angebotes Verlässlichkeit und Kontinuität zu gewährleistet. Außerhalb der Öffnungszeiten der Tagesklinik bedarf es im Akutfall einer klaren Zuständigkeit durch die versorgende stationäre KJP. In einem weiteren Schritt könnten wie von Keilen [2] empfohlen, in den dezentralen Tageskliniken wechselnde Bereitschaftsdienste für Nachtstunden und Wochenenden mit einer geringen Krisenbettenanzahl angedacht werden.

Für die organisatorische Abstimmung hinsichtlich patienten:innenbezogener Angebote mit teambezogenen Elementen braucht es eine Ausarbeitung konkreter Wochenpläne. Diese sollen einerseits eine klare zeitliche Struktur zur Orientierung für Patient:innen und Personal zur Verfügung stellen. Andererseits sollten sie genug Spielraum für milieutherapeutische Prozesse offenhalten, sowie eine individuell angepasste und an Leitlinien orientierte Behandlungsplanung für die einzelnen Patient:innen ermöglichen. Zeitfenster für notwendige Vernetzungstätigkeit müssen mitberücksichtigt werden.

In der konkreten Ausgestaltung tagesklinischer Einrichtungen sollten regionale Besonderheiten berücksichtigt und Kooperation mit räumlich nahen Einrichtungen, wie Schulen und Freizeiteinrichtungen in unmittelbarer Nähe gesucht werden, um die Integration zwischen tagesklinischem Setting und sozialem Umfeld zu fördern [2].

Neben Tageskliniken mit einem allgemeinen Versorgungsanspruch können je nach regionalem Bedarf und Möglichkeit tagesklinische Gruppen mit spezifischen Konzepten und Spezialaufträgen entwickelt werden. Beispiele hierfür sind Tagekliniken für Transitionspsychiatrie (welche in Österreich noch deutliche Defizite aufzeigt) [20], Kleinkindertageskliniken (zur Entwicklungsförderung) und kooperative Tagesklinik (die in engem Schulterschluss mit der Stammschule und der Heilstättenschule operiert) wie derzeit zum Beispiel beide am Standort Hinterbrühl/NÖ umgesetzt werden, oder eine Eltern-Kind-Tagesklinik (wie sie bereits in Deutschland etabliert) [21, 22]. Weiters könnte es zu einer Kombination bzw. engen Zusammenarbeit zwischen Tageskliniken und der Kinder- und Jugendhilfe kommen (wie bereits in der „Tagesklinik Extended Soulspace“ in Wien, PSD etabliert) [23].
